# Spirooxindole Derivative SOID-8 Induces Apoptosis Associated with Inhibition of JAK2/STAT3 Signaling in Melanoma Cells

**DOI:** 10.1371/journal.pone.0049306

**Published:** 2012-11-16

**Authors:** Yan Tian, Sangkil Nam, Lucy Liu, Fumiko Yakushijin, Kenichi Yakushijin, Ralf Buettner, Wei Liang, Fan Yang, Yuelong Ma, David Horne, Richard Jove

**Affiliations:** Department of Molecular Medicine, Beckman Research Institute, City of Hope Comprehensive Cancer Center, Duarte, California, United States of America; University of Tennessee, United States of America

## Abstract

Melanoma is generally refractory to current chemotherapy, thus new treatment strategies are needed. In this study, we synthesized a series of spirooxindole derivatives (SOID-1 to SOID-12) and evaluated their antitumor effects on melanoma. Among the 12 spirooxindole derivatives, SOID-8 showed the strongest antitumor activity by viability screening. SOID-8 inhibited viability of A2058, A375, SK-MEL-5 and SK-MEL-28 human melanoma cells in a dose- and time-dependent manner. SOID-8 also induced apoptosis of these tumor cells, which was confirmed by positive Annexin V staining and an increase of poly(ADP-ribose) polymerase cleavage. The antiapoptotic protein Mcl-1, a member of the Bcl-2 family, was downregulated and correlated with SOID-8 induced apoptosis. In addition, SOID-8 reduced tyrosine phosphorylation of Signal Tansducer and Activator of Transcription 3 (STAT3) in both dose- and time-dependent manners. This inhibition was associated with decreased levels of phosphorylation of Janus-activated kinase-2 (JAK2), an upstream kinase that mediates STAT3 phosphorylation at Tyr705. Accordingly, SOID-8 inhibited IL-6-induced activation of STAT3 and JAK2 in melanoma cells. Finally, SOID-8 suppressed melanoma tumor growth in a mouse xenograft model, accompanied with a decrease of phosphorylation of JAK2 and STAT3. Our results indicate that the antitumor activity of SOID-8 is at least partially due to inhibition of JAK2/STAT3 signaling in melanoma cells. These findings suggest that the spirooxindole derivative SOID-8 is a promising lead compound for further development of new preventive and therapeutic agents for melanoma.

## Introduction

Melanoma, the most dangerous form of skin cancer, has increased rapidly in developed countries during the past several decades [1]. Early removal of melanoma lesions leads to a good prognosis, but once it has progressed to the metastatic stage, it is extremely difficult to treat and largely refractory to existing therapies, with median survival time only 6–9 months and a 3-year survival rate of only 10–15% [2], [3]. Over the last 40 years, no single drug or combination of drugs demonstrated any impact on survival of metastatic melanoma [4], [5]. An understanding of the mechanisms responsible for melanoma initiation, progression and maintenance is critical for developing successful therapies, and recent developments in the area of immunotherapy as well as targeted therapy for melanoma are promising [6], [7]. An anti-CTLA-4 monoclonal antibody (ipilimumab) and a BRAF inhibitor (vemurafenib) were approved in 2011 by FDA to treat advanced melanoma, which herald a new era of targeted therapeutics for melanoma [8], [9].

Signal Transducer and Activator of Transcription 3 (STAT3) is a transcriptional factor that is activated by many cytokines and growth factors, and plays a key role in cell survival, proliferation, and differentiation [10], [11]. Under normal physiological conditions, the activation of STAT3 is tightly regulated and occurs transiently. In cancers, STAT3 becomes activated constitutively through aberrant activation of tyrosine kinases, such as c-Src or Janus kinase (JAK) family members, thereby driving the malignant phenotype of cancer cells, including melanoma. STAT3 activation requires Tyr705 phosphorylation, resulting in dimerization, nuclear translocation, DNA binding, and transcriptional activation of target genes. STAT3 regulates basic biologic processes important in tumorigenesis including cell cycle progression, survival, tumor angiogenesis, and tumor-cell evasion of the immune system [10], [11]. Many of the studies that defined the role of STAT3 in oncogenesis were carried out in cancer cells and animal models of melanoma, and targeting STAT3 signaling in melanoma cells is an appealing strategy [12], [13]. Recently, small-molecule inhibitors of the STAT3 signaling have been reported to be effective anticancer agents *in vitro* and *in vivo*, indicating promising targeted therapies using molecules for cancers are emerging [14]–[18].

Natural products or synthetic compounds inspired from natural products continue to be excellent sources for new drug candidates, especially in the area of anticancer therapeutics [19]. The 3,3′-pyrrolidinyl-spirooxindole unit is a heterocyclic motif that forms the core of a large family of alkaloid natural products with strong bioactivity profiles and interesting structural properties. Significant recent advances in the synthesis of this fused heterocyclic system have led to intense interest in the development of related compounds as potential medicinal agents or biological probes [20]–[22].

Here we report that SOID-8, a derivative of spiro[pyrrolidin-3,3′-oxindole], inhibits growth and induces apoptosis of melanoma cells. The anticancer effects of this compound are associated with suppression of phosphorylated JAK2 and STAT3. Importantly, SOID-8 inhibits melanoma tumor growth in a mouse xenograft model. Together, these findings not only demonstrate that SOID-8 blocks the JAK2/STAT3 signaling *in vitro* and *in vivo*, but also provide a potential novel lead compound for further development of new preventive and therapeutic agents for melanoma.

## Results

### Spirooxindole derivatives inhibit viability of melanoma cells

**Figure 1:**
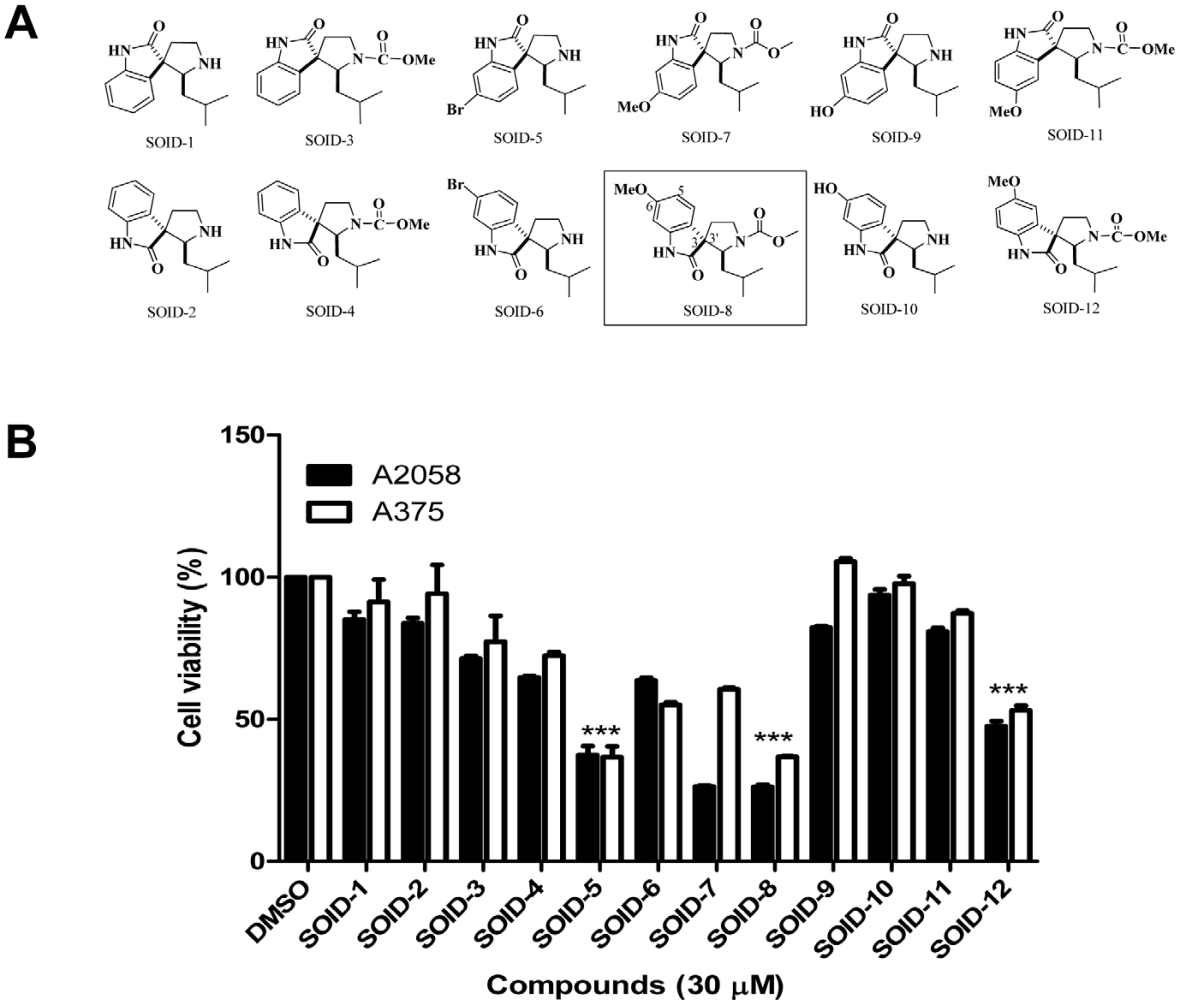
Screening of spirooxindole derivatives for anti-tumor activity on melanoma cells. (A) Structure of analogues SOID-1 to SOID-12 inspired by natural product Sprotryprostatin B. (B) Effect of spirooxindole derivatives on melanoma cell growth. A2058 and A375 cells were treated with a series of spirooxindole derivatives (30 µM) for 24 h, and cell viability was evaluated by MTS assay. DMSO was used as vehicle control. Data are shown as means ± SEM. Statistical significance between SOID-8 treatment and DMSO control, determined by the two-tailed Student's t test, is indicated by ***, *p*<0.001.

Twelve analogues of the natural product Spirotryprostatin B, named SOID-1 to SOID-12, were synthesized (Fig. 1A) [23]. These spirooxindole derivatives represent 6 pairs of diastereoisomers. We determined their effects on the viability of melanoma cells by using MTS cell-viability assays at a concentration of 30 µM. After 24 h treatment, SOID-5, 8 and SOID-12 showed significant inhibition of viability in both A2058 and A375 cells with the *p* values were less than 0.001 by statistical analysis (Fig. 1B). Since SOID-8 displayed more potent anticancer activity than other spirooxindole derivatives in both melanoma cell lines, it was chosen for further study.

### SOID-8 inhibits viability of melanoma cells

**Figure 2:**
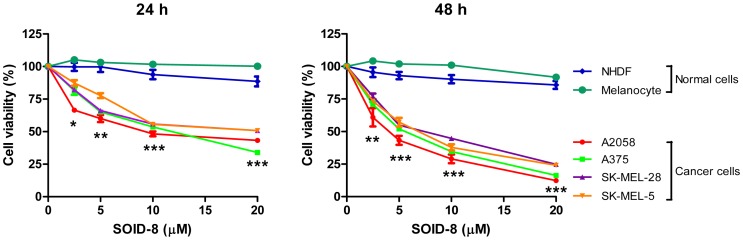
SOID-8 inhibits melanoma cell growth. (**A**) SOID-8 significantly inhibits viability of human melanoma cell lines (A2058, A375, SK-MEL-28 and SK-MEL-5), but has minor effect on normal human cells [NHDFs (normal human dermal fibroblasts) and melanocytes]. Cells were treated with increasing concentrations (2.5, 5, 10, 20 µM) of SOID-8 for 24 (left) and 48 h (right), and cell viability was evaluated by MTS assay. DMSO was used as vehicle control. Data are shown as means ± SEM. Statistical significance between any one of two normal cell lines (NHDF or melanocyte) and any one of four melanoma cell lines (A2058, A375, SK-MEL-28, or SK-MEL-5) was determined by the two-tailed Student's t test. *, *p*<0.05; **, *p*<0.005; ***, *p*<0.001.

To further evaluated the effects of SOID-8 on the viability of melanoma cells, A2058, A375, SK-MEL-28 and SK-MEL-5 human melanoma cells were treated with increasing concentrations of SOID-8 (2.5, 5, 10, 20 µM) for 24 and 48 h, respectively. As shown in Figure 2, cell viability of all four melanoma cell lines was inhibited by SOID-8 in a dose- and time-dependent manner. A2058 and A375 cells were more sensitive to SOID-8 treatment than SK-MEL-28 and SK-MEL-5 cells. The 50% inhibitory concentrations (IC_50_) of SOID-8 in A2058 cells were 9.3 µM at 24 h and 3.7 µM at 48 h. The IC_50_ of SOID-8 in A375 cells were 11.85 µM at 24 h and 5.3 µM at 48 h. By contrast, SOID-8 had very little effect on the growth of normal human dermal fibroblasts (NHDFs) and human melanocytes (Fig. 2). Moreover, the growth of all four melanoma cell lines was significantly inhibited by SOID-8 when compared to that of normal NHDFs and melanocytes. These results demonstrate that SOID-8 inhibits cell viability of human melanoma cells, without significant cytotoxicity to normal human cells *in vitro*.

### SOID-8 induces apoptosis and downregulates Mcl-1 in melanoma cells

**Figure 3:**
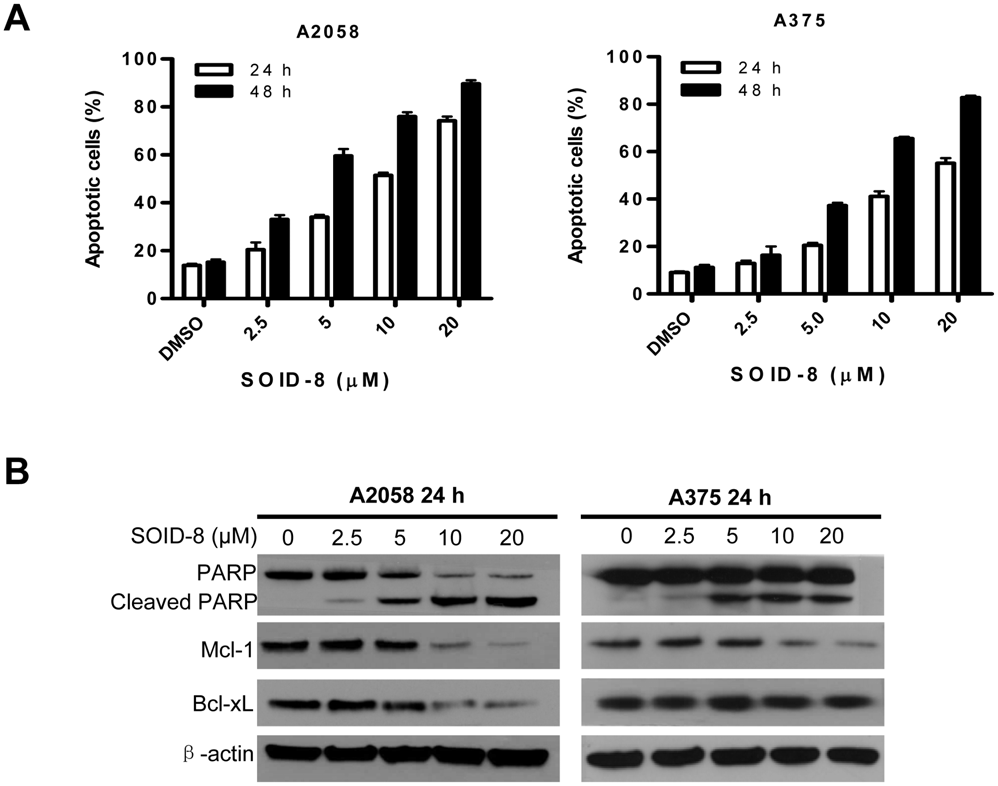
Effects of SOID-8 on apoptosis of melanoma cells. (**A**) SOID-8 induces apoptosis of A2058 and A375 cells. Cells were treated with SOID-8 at indicated concentrations for 24 (left) and 48 h (right), respectively. Apoptotic cells are represented by propidium iodide and Annexin V-FITC double-positive staining as determined by fluorescence-activated cell sorting. Each experiment was done in triplicate and repeated twice independently. (**B**) Effects of SOID-8 on apoptosis-related proteins. A2058 and A375 cells were treated with increasing concentrations of SOID-8 for 24 h and the level of PARP, Mcl-1 and Bcl-xL protein was measured by western blot.

Next, we investigated whether SOID-8 induced apoptosis of melanoma tumor cells. A2058 and A375 cells were treated with increasing concentrations of SOID-8 (2.5, 5, 10, 20 µM) for 24 and 48 h, and both attached and floating cells were collected and analyzed by Annexin V-FITC/PI staining and fluorescence-activated cell sorting. Apoptotic cells, including both early apoptotic cells (Annexin V positive) and late double-positive apoptotic cells (Annexin V and PI positive), were increased by SOID-8 treatment in a dose- and time-dependent manner (Fig. 3A). These results indicate that SOID-8 can effectively induce apoptosis of human melanoma cells.

Poly(ADP-ribose) polymerase (PARP) is essential for cell survival; it is cleaved during apoptosis and loses function. To further confirm that the cell death induced by SOID-8 was an apoptotic process, we assayed expression of cleaved PARP after SOID-8 treatment. Both A2058 and A375 cells were treated with SOID-8 or vehicle for 24 h, then the levels of cleaved PARP were detected by immunoblotting assays. SOID-8 increased cleaved PARP in both melanoma cell lines (Fig. 3B). As low as 5 µM SOID-8 strongly increased the cleavage of PARP in these cells.

Bcl-2 family proteins are important regulators of apoptosis [24]. To determine whether SOID-8-induced apoptosis of A2058 and A375 cells is due to down-regulation of the antiapoptotic proteins in this family, the levels of Mcl-1 and Bcl-xL were examined after 24 h SOID-8 treatment. Although Bcl-xL was only decreased in A2058, Mcl-1 was decreased in both melanoma cell lines (Fig. 3B). These results are consistent with the induction of apoptosis by SOID-8 and implicate the importance of Mcl-1 in this response.

### SOID-8 reduces phosphorylation of STAT3 and JAK2 in A2058 and A375 cells

Activation of STAT3 signaling has an important role in melanoma oncogenesis [13]. To investigate whether SOID-8 can inhibit the STAT3 signaling pathway in human melanoma cells, we examined the levels of total and phosphorylated STAT3 in A2058 and A375 melanoma cells after 24 h SOID-8 treatment. We found that phosphorylation of STAT3 at Tyr705 was significantly inhibited in a dose-dependent manner in both A2058 and A375 cells after 24 h SOID-8 treatment, whereas total protein levels of STAT3 were not significantly changed with SOID-8 treatment.

**Figure 4:**
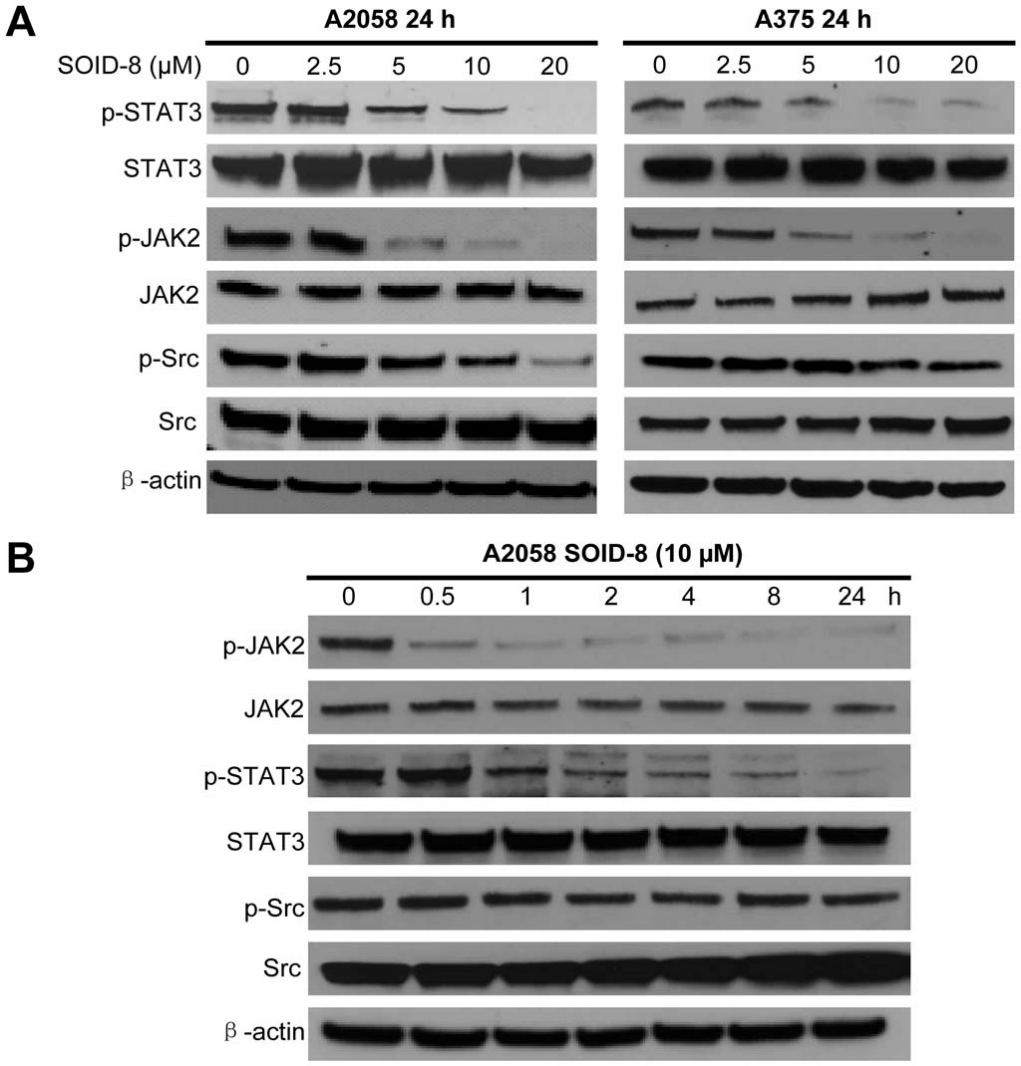
SOID-8 represses the JAK2/STAT3 signaling pathway in a dose- and time-dependent manner. (**A**) Total proteins were isolated from A2058 (left) and A375 (right) cells incubated with 2.5, 5, 10, or 20 µM SOID-8 for 24 h. Western blot was done with antibodies to total or phosphorylated (*p*) STAT3, JAK2 and Src using 40 µg total proteins. β-Actin was used as a loading control. (B) Time course of inhibition of STAT3 upstream regulatory proteins JAK2 following SOID-8 treatment. A2058 cells were treated with 10 µM of SOID-8 for indicated times. Total proteins were isolated and the level of total or phosphorylated (*p*) STAT3, JAK2 and Src was measured by western blot.

Constitutive activation of STAT3 induced by Src and JAK tyrosine kinases participates in growth regulation of tumor cells [10]. To further elucidate how SOID-8 suppresses phophorylation of STAT3 at Tyr705, expression of total and phosphorylated of JAK2 and c-Src proteins were examined after 24 h SOID-8 treatment. As shown in Fig. 4B, SOID-8 treatement resulted in a reduction of phosphorylation of JAK2 in a dose-dependent manner in both A2058 and A375 cells. Although phosphorylation of Src was repressed in A2058 cells at 10 µM of SOID-8, no significant inhibitory effects were observed in A375 cells. To ascertain the length of treatment time required for SOID-8 to suppress phosphotyrosine STAT3 and JAK2 levels in A2058 cells, we carried out a time course experiment (Fig. 4B). Suppression of p-JAK2 and p-STAT3 by SOID-8 in melanoma cells was observed in a time-dependent manner. Inhibition of phosphorylation of JAK2 was detected at 0.5 h post SOID-8 treatment, while inhibition of phosphorylated STAT3 was detected at 2 h post SOID-8 treatment. These results are consistent with that JAK2 as an upstream regulator of STAT3. Taken together, our data indicate that SOID-8 inhibits the JAK2/STAT3 signaling pathway in melanoma cells, associated with induction of apoptosis induced by SOID-8.

### SOID-8 suppresses IL-6-induced STAT3 and JAK2 phosphorylation of melanoma cells

**Figure 5:**
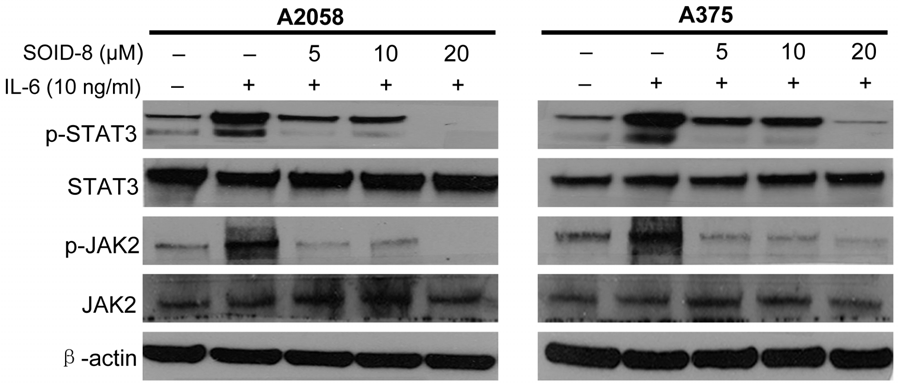
SOID-8 inhibits IL-6-induced phosphorylated STAT3 and JAK2 in A2058 (left) and A375 (right) cells. The cells were serum-starved overnight, then left untreated or were treated with SOID-8 (5–20 µM) for 24 h. The untreated and SOID-8-treated cells were stimulated with IL-6 (10 ng/mL). Cells were then harvested after 30 minutes and the level of total or phosphorylated (*p*) STAT3 and JAK2 was analyzed by western blot.

Interleukin-6 (IL-6) is a critical growth factor for melanoma cells, IL-6 can directly activate STAT3 phosphorylation through JAK family kinases [10], [25]. To determine whether SOID-8 inhibits phosphorylation of STAT3 and JAK2 by IL-6 stimulation, serum-free cultured A2058 and A375 cells were treated with SOID-8 at the concentrations indicated (5, 10, 20 µM) for 24 h, and then IL-6 (10 ng/mL) was added to cells for 30 min. Immunoblotting assays showed that SOID-8 inhibited the phosphorylation of STAT3 and JAK2 induced by IL-6 in both cell lines (Fig. 5).

### SOID-8 inhibits human melanoma tumor growth in a mouse xenograft model

**Figure 6:**
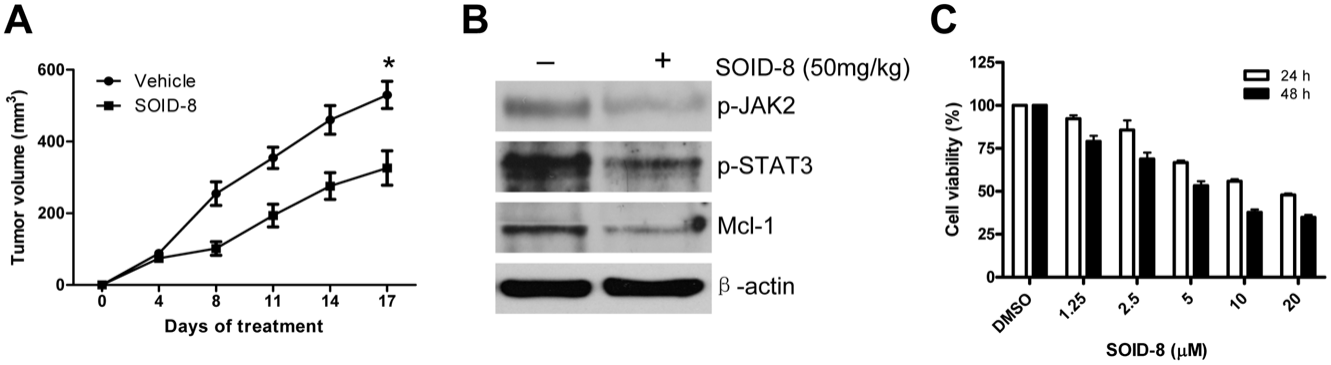
SOID-8 inhibits melanoma tumor growth in a mouse xenograft model. (A) SOID-8 suppressed tumor growth of A2058 melanoma xenografts. A2058 cells (2.5×106) were implanted subcutaneously into the flanks of NSG mice. After tumor development (one week later), SOID-8 or vehicle control was administered by oral gavage twice a day at 50 mg/kg for 17 days. Data are mean ± SEM. Statistical significance, determined by the two-tailed ANOVA, is indicated by *, *p*<0.01, n = 8 mice/group. (**B**) SOID-8 inhibits the level of phospho-JAK2 and phospho-STAT3, and Mcl-1 in A2058 tumors, as determined by western blot analysis. (**C**) SOID-8 inhibits viability of B16 mouse melanoma cells. Cells were treated with increasing concentrations of SOID-8 (1.25, 2.5, 5, 10, or 20 µM) for 24 and 48 h, and cell viability was evaluated by MTS assay. DMSO was used as vehicle control.

To examine the effect of SOID-8 on growth of human A2058 melanoma cells *in vivo*, we first tested the toxicity of SOID-8 in BALB/c normal mice. At 200 mg/kg, SOID-8 was found to be non-toxic in mice (data not shown). We then used 50 mg/kg for a SOID-8 therapeutic study in an A2058 xenograft model using NSG mice. SOID-8 was administered orally, twice a day for 17 days. No lethal toxicity or weight loss was observed among the tested mice. As shown in Fig. 6A, significant tumor growth inhibition was observed in the SOID-8 group (n = 8). The trends of tumor growth curves in control and treated groups were similar after 8 days of treatment. However, tumors in treated group always grew slower than ones in the control group. Importantly, the size of tumors in the treated group with SOID-8 was inhibited approximately 40% compared with controls at 17 days after treatment. Consistent with the observations *in vitro*, SOID-8 reduced phosphorylated JAK2 and STAT3, and inhibited Mcl-1 protein expression *in vivo* (Fig. 6B). Thus, these data demonstrate the antitumor activity of SOID-8 *in vivo* against human melanoma cells. To determine whether SOID-8 also inhibits proliferation of mouse melanoma cells, we examined the effect of SOID-8 on B16 mouse melanoma cells. Our results showed that SOID-8 could inhibit viability of B16 mouse melanoma cells in a time- and dose-dependent manner (Fig. 6C).

## Discussion

Synthetic or natural heterocyclic compounds, particularly spirooxindoles with a spirocyclic quaternary stereocenter at the C3 position, are endowed with a wide range of pharmacological activities [26], [27]. For example, oxindole-type phytoalexins from plants have shown potent antimicrobial, antitumor, and oviposition-stimulant biological activities [28], but relatively few studies are concerned with the bioactive mechanism studies of these compounds. There is considerable interest for drug-lead synthesis by highly efficient synthetic methods to access optically active spriooxindole, and to provide a foundation for further development of new types of therapeutic agents through preliminary biological studies [29]–[31]. Here, we screened twelve compounds (6 diastereoisomer pairs of spirooxindole derivatives) on melanoma cells by MTS cell-viability assays, and the results showed that SOID-8 is a more potent compound in melanoma cells. Comparing the structures and activities of these compounds, we found that the diastereoisomer relationship is not a major factor in terms of antitumor activity. The 6-substitution but not 7-substitution in the aromatic ring is important to improve the anticancer activity, and the bromo- and methoxy-substitution are more potent than the hydroxyl-substitution. These results indicate the modification on the aromatic ring at the 6-position is important to improve anticancer activity and provide us some clues for further rational development derivatives.

Since existing therapies do not greatly improve the survival rate of advanced melanoma, it is critical to find new treatments based on a better understanding of the molecular basis of malignant progression for this tumor. In our study, SOID-8 has shown induction of apoptosis of melanoma cells both *in vitro* and *in vivo*. The antitumor activity of SOID-8 is associated with inhibition of JAK2/STAT3 signaling in these tumor cells. In a majority of melanoma cell lines and clinical specimens, constitutive phosphorylation of STAT3 at the Tyr705 residue has been observed, and this post-translational modification occurs in response to a variety of cytokines such as IL-6 (interleukin-6) which can be secreted in an autocrine fashion by melanoma cells, and is mediated by JAK2 kinase. Recent data suggests that inhibition of the JAK/STAT3 pathway is a potential therapeutic approach for melanoma [13]. Our data shows that SOID-8 reduced tyrosine phosphorylation of STAT3 in both dose- and time-dependent manners, and this suppression was mediated through decreased levels of phosphorylation of JAK2 but not c-Src in A2058 and A375 melanoma cells. Significantly, SOID-8 inhibits IL-6-induced STAT3 and JAK2 activation in melanoma cells, further supporting this notion.

Consistent with the induction of apoptosis by SOID-8, the antiapoptotic protein Mcl-1, downregulated by SOID-8 in both A2058 and A375 cells, implicated the importance of Mcl-1 in this response. Mcl-1 is a member of the Bcl-2 family of proteins, which has been shown to play a critical role in the survival of malignant cells, including melanoma [32], [33]. Blocking STAT3 protein in human tumor cells has been shown to down-regulate Mcl-1 expression and induce tumor cell apoptosis [34], [35]. STAT3 and Mcl-1 are the proteins inhibited in common among A2058 and A375 cells. Therefore, down-regulation of Mcl-1 by inhibition of phosphorylated STAT3 may be an important mechanism of action of SOID-8 in these melanoma cells. Several small molecules have been identified as lead compounds that block STAT3 signaling, althourgh they remain to be further developed in terms of potency and specificity [36], [37].

Importantly, SOID-8 suppressed the tumor growth of human melanoma in a mouse xenograft model. Furthermore, phosphorylation of JAK2 and STAT3, as well as Mcl-1, was downregulated in SOID-8-treated xenograft tumors compared to control tumors. These results further confirm that antitumor activity of SOID-8 is at least partially due to inhibition of JAK2/STAT3 signaling in human melanoma cells. Of note, the solubility of SOID-8 is low in 30% solutol solution at the concentration is of 10 mg/ml, and we thus conjecture that the solubility and absorbability of SOID-8 may impair its antitumor effect in the *in viro* mouse studies. To improve the activity and solubility of SOID-8, more derivatives must be synthesized for further study.

In this study, we have discovered that the spirooxindole derivative SOID-8 inhibits melanoma cell proliferation and induces apoptosis of melanoma cells at least in part due to inhibition of the JAK2/STAT3 signaling pathway, *in vitro* and *in vivo*. These findings for the first time provide evidence of a novel mechanism for spirooxindole derivatives's antitumor activity. Our results suggest that SOID-8 is a promising lead compound for further development of new preventive and therapeutic agents for melanoma.

## Materials and Methods

### Reagents

Twelve spirooxindole derivatives were synthesized as described previously [23], which represent 6 pairs of diastereoisomers. Compounds SOID-9 and SOID-10 are natural spirooxindole derivatives, called isoelacomine and elacomine, respectively. Others are novel spirooxindole derivatives and differ by position of substitution on the aromatic ring. All compounds were prepared in dimethyl sulfoxide (DMSO) at a concentration of 40 mM and stored as small aliquots at -20°C. For *in vivo* mouse experiments, SOID-8 was dissolved in 30% Solutol (BASF) at a concentration of 10 mg/mL. Bacteria-derived recombinant human IL-6 was purchased form R&D Systems. Anti-β-actin (Cat#A1978) was purchased from Sigma. Polyclonal antibodies to phospho-Stat3 (Tyr705) (Cat#9131), Stat3 (Cat#9132), phospho-Jak2 (Tyr1007/1008) (Cat#3771), Jak2 (Cat#3230), phosphor-Src family (Tyr416) (Cat#2101), Src (Cat#2108), Mcl-1 (Cat #4572), PARP (Cat#9542), Bcl-xL (Cat#2762) were obtained from Cell Signaling Technology (Danvers, MA). Horseradish peroxidase (HRP)-labeled anti-mouse and anti-rabbit secondary antibodies were obtained from GE Healthcare.

### Cell culture

The human A2058, A375, SK-MEL-5, SK-MEL-28 and mouse B16 melanoma cell lines were obtained from the American Type Culture Collection. NHDFs (normal human dermal fibroblasts) were a gift from Dr. Jun Wu (Division of Comparative Medicine, City of Hope), which were purchased from Lonza. All melanoma cells were maintained in RPMI-1640 medium and NHDFs were maintained in Dulbecco's Modified Eagle's Medium (DMEM) supplemented with 10% FBS (fetal bovine serum, Sigma) and 1% Antibiotic-Antimycotic (Gibco). Human melanocytes were purchased from Invitrogen and maintained following the supplier's instructions. All cultured cells were grown in a humidified atmosphere of 5% CO_2_ at 37°C.

### Cell-viability assay

Cell-viability assays were performed with CellTiter 96 Aqueous One Solution Cell Proliferation Assay (Promega), which contains 3-(4,5-dimethylthiazol-2-yl)-5-(3-carboxymethoxyphenyl)-2-(4-sulfophenyl)-2H-tetrazolium (MTS). Human cancer cells were seeded onto 96-well plates at a density of 5,000 cells per well in culture medium with 10% FBS. After overnight culture (16 h), the cells were treated with compounds at the concentrations indicated or with DMSO as the vehicle control. After 24 or 48 h treatment, MTS was added to the cells according to the manufacturer's instructions. Within 2 h absorbance was measured at 490 nm using an automated ELISA plate reader (Bio-Rad). The values of cell viability were calculated as percentages of absorbance from the treated samples to absorbance of the vehicle control.

### Apoptosis assay

A2058 and A375 melanoma cells (2×10^5^) were seeded on 60 mm culture dishes in culture medium contains 10% FBS. The following day the cells were treated with the indicated concentrations of SOID-8 for 24 or 48 h. After treatment, all cells, including both floating and attached cells, were collected. The apoptotic cells were detected by fluorescence-activated cell sorting with an Annexin V-FITC Apoptosis Detection Kit (BD Biosciences) according to the supplier's protocol, in which the early- and late-death cells were stained with Annexin V-FITC and PI (propidium iodide).

### Western blot analysis

To detect various proteins, A2058 and A375 cells were treated with SOID-8 for the indicated times. Protein lysates were prepared in RIPA buffer with protease and phosphotase inhibitors (Thermo). Protein concentrations were determined by BioMate Spectrometer (Thermo) and protein assay (Bio-Rad). Total proteins (40 µg) were resolved in 4–20% gradient Tris-HEPES gels (Thermo). After gel electrophoresis, the proteins were transferred to Hybond-C membranes (Amersham). The membranes were blocked in 5% nonfat milk in 1× PBS with 0.1% Tween 20 (PBST) at room temperature for 1–2 hours, followed by an overnight incubation at 4°C with primary antibodies in 5% non-fat milk PBST solution. The membranes were then washed with PBST and incubated with HRP-conjugated anti-mouse or anti-rabbit secondary antibodies for 1–2 hours at room temperature, or overnight at 4°C. Immunoreactivity was detected with SuperSignal West Pico Chemiluminescent or Dura Extended Duration Substrate (Pierce).

### Tumor xenografts and drug administration

BALB/c mice (6–8 weeks old, for toxicity tolerance studies) were obtained from National Cancer Institute. Immunodeficient NOD/SCID/IL2Rgamma null (NSG) mice (female; 7–8 weeks old) were purchased from The Jackson Laboratory for use as the xenograft model. Animal use procedures were approved by the Institutional Animal Care and Use Committee (IACUC) of the Beckman Research Institute at City of Hope Medical Center. For the subcutaneous xenograft model, 2.5×10^6^ A2058 human melanoma cells suspended in serum-free medium were injected into the flank of NSG mice. When tumors became palpable after one week development, animals were randomized into 8 mice per group and SOID-8 or vehicle was administered via oral gavage twice a day at the dose of 50 mg/kg body weight. Tumor growth was monitored every other day, and tumor size was measured every 3 to 4 days by caliper. Tumor volumes were calculated by the formula: 0.5× (larger diameter)×(small diameter)^2^.

### Statistical analysis

A two-tailed *t* test was used to evaluate statistical significance of differences between treated and control groups. ***, *p*<0.001, **, *p*<0.005 and *, *p*<0.05.

## References

[1] Berwick M, Erdei E, Hay J (2009) Melanoma epidemiology and public health. Dermatol Clin 27: 205–214. 19254665 10.1016/j.det.2008.12.002 PMC3595561

[2] Gray-Schopfer V, Wellbrock C, Marais R (2007) Melanoma biology and new targeted therapy. Nature 445: 851–857. 17314971 10.1038/nature05661

[3] Chin L, Garraway LA, Fisher DE (2006) Malignant melanoma: genetics and therapeutics in the genomic era. Genes Dev 20: 2149–2182. 16912270 10.1101/gad.1437206

[4] Balch CM, Gershenwald JE, Soong SJ, Thompson JF, Atkins MB, et al (2009) Final version of 2009 AJCC melanoma staging and classification. J Clin Oncol 27: 6199–6206. 19917835 10.1200/JCO.2009.23.4799 PMC2793035

[5] Eggermont AM, Robert C (2011) New drugs in melanoma: it's a whole new world. Eur J Cancer 47: 2150–2157. 21802280 10.1016/j.ejca.2011.06.052

[6] Ko JM, Fisher DE (2011) A new era: melanoma genetics and therapeutics. J Pathol 223: 241–250. 21125678 10.1002/path.2804

[7] Ji Z, Flaherty KT, Tsao H (2010) Molecular therapeutic approaches to melanoma. Mol Aspects Med 31: 194–204. 20176049 10.1016/j.mam.2010.02.004

[8] Mellman I, Coukos G, Dranoff G (2011) Cancer immunotherapy comes of age. Nature 480: 480–489. 22193102 10.1038/nature10673 PMC3967235

[9] Finn L, Markovic SN, Joseph RW (2012) Therapy for metastatic melanoam: the past, present, and future. BMC Med 10: 23–32. 22385436 10.1186/1741-7015-10-23 PMC3308914

[10] Yu H, Jove R (2004) The STATs of cancer–new molecular targets come of age. Nat Rev Cancer 4: 97–105. 14964307 10.1038/nrc1275

[11] Yu H, Pardoll D, Jove R (2009) STATs in cancer inflammation and immunity: a leading role for STAT3. Nat Rev Cancer 9: 798–809. 19851315 10.1038/nrc2734 PMC4856025

[12] Gao SP, Bromberg JF (2006) Touched and moved by STAT3. Sci STKE 2006: pe30. 16835434 10.1126/stke.3432006pe30

[13] Kortylewski M, Jove R, Yu H (2005) Targeting STAT3 affects melanoma on multiple fronts. Cancer Metastasis Rev 24: 315–327. 15986140 10.1007/s10555-005-1580-1

[14] Page BD, Ball DP, Gunning PT (2011) Signal transducer and activator of transcription 3 inhibitors: a patent review. Expert Opin Ther Pat 21: 65–83. 21114420 10.1517/13543776.2011.539205

[15] Sun J, Blaskovich MA, Jove R, Livingston SK, Coppola D, et al (2005) Cucurbitacin Q: a selective STAT3 activation inhibitor with potent antitumor activity. Oncogene 24: 3236–3245. 15735720 10.1038/sj.onc.1208470

[16] Blaskovich MA, Sun J, Cantor A, Turkson J, Jove R, et al (2003) Discovery of JSI-124 (cucurbitacin I), a selective Janus kinase/signal transducer and activator of transcription 3 signaling pathway inhibitor with potent antitumor activity against human and murine cancer cells in mice. Cancer Res 63: 1270–1279. 12649187

[17] Nam S, Buettner R, Turkson J, Kim D, Cheng JQ, et al (2005) Indirubin derivatives inhibit Stat3 signaling and induce apoptosis in human cancer cells. Proc Natl Acad Sci USA 102: 5998–6003. 15837920 10.1073/pnas.0409467102 PMC1087919

[18] Liu L, Nam S, Tian Y, Yang F, Wu J, et al (2011) 6-Bromoindirubin-3′-oxime inhibits JAK/STAT3 signaling and induces apoptosis of human melanoma cells. Cancer Res 71: 3972–3979. 21610112 10.1158/0008-5472.CAN-10-3852 PMC3107399

[19] Paterson I, Anderson E (2005) The renaissance of natural products as drug candidates. Science 21: 451–453. 10.1126/science.1116364 16239465

[20] Galliford CV, Scheidt KA (2007) Pyrrolidinyl-spirooxindole natural products as inspirations for the development of potential therapeutic agents. Angew Chem Int Ed Engl 46: 8748–8758. 17943924 10.1002/anie.200701342

[21] Chowdhury S, Chafeev M, Liu S, Sun J, Raina V, et al (2011) Discovery of XEN907, a spirooxindole blocker of NaV1.7 for the treatment of pain. Bioorg Med Chem Lett 21: 3676–3681. 21570288 10.1016/j.bmcl.2011.04.088

[22] Bhaskar G, Arun Y, Balachandran C, Saikumar C, Perumal PT (2012) Synthesis of novel spirooxindole derivatives by one pot multicomponent reaction and their antimicrobial activity. Eur J Med Chem 51: 79–91. 22405285 10.1016/j.ejmech.2012.02.024

[23] Miyake FY, Yakushijin K, Horne DA (2004) Preparation and synthetic applications of 2-halotryptophan methyl esters: synthesis of spirotryprostatin B. Angew Chem Int Ed Engl 40: 5357–60. 10.1002/anie.200460419 15468070

[24] Maddika S, Ande SR, Panigrahi S, Paranjothy T, Weglarczyk K (2007) Cell survival, cell death and cell cycle pathways are interconnected: implications for cancer therapy. Drug Resist Updat 10: 13–29. 17303468 10.1016/j.drup.2007.01.003

[25] Sansone P, Bromberg J (2012) Targeting the interleukin-6/jak/stat pathway in human malignancies. J Clin Oncol 30: 1005–1014. 22355058 10.1200/JCO.2010.31.8907 PMC3341105

[26] Dounay AB, Hatanaka K, Kodanko JJ, Oestreich M, Overman LE, et al (2003) Catalytic asymmetric synthesis of quaternary carbons bearing two aryl substituents. Enantioselective synthesis of 3-alkyl-3-aryl oxindoles by catalytic asymmetric intramolecular heck reactions. J Am Chem Soc 125: 6261–6271. 12785859 10.1021/ja034525d

[27] Liu YL, Zhou F, Cao JJ, Ji CB, Ding M (2010) A facile method for the synthesis of oxindole based quaternary alpha-aminonitriles via the Strecker reaction. Org Biomol Chem 8: 3847–3850. 20617240 10.1039/c0ob00174k

[28] Galliford CV, Scheidt KA (2007) Pyrrolidinyl-Spirooxindole natural products as inspirations for the development of potential therapeutic agents. Angew Chem Int Ed Engl 46: 8748–58. 17943924 10.1002/anie.200701342

[29] Jiang X, Cao Y, Wang Y, Liu L, Shen F (2010) A unique approach to the concise synthesis of highly optically active spirooxazolines and the discovery of a more potent oxindole-type phytoalexin analogue. J Am Chem Soc 132: 15328–15333. 20939568 10.1021/ja106349m

[30] Chen T, Xu XP, Ji SJ (2010) Novel, one-pot, three-component route to indol-3-yl substituted spirooxindole derivatives. J Comb Chem 12: 659–663. 20593846 10.1021/cc100064c

[31] Chen XH, Wei Q, Luo SW, Xiao H, Gong LZ (2009) Organocatalytic synthesis of spiro[pyrrolidin-3,3′-oxindoles] with high enantiopurity and structural diversity. J Am Chem Soc 131: 13819–13825. 19736987 10.1021/ja905302f

[32] Lessene G, Czabotar PE, Colman PM (2008) BCL-2 family antagonists for cancer therapy. Nat Rev Drug Discov 7: 989–1000. 19043450 10.1038/nrd2658

[33] Senft D, Berking C, Graf SA, Kammerbauer C, Ruzicka T, et al (2012) Selective induction of cell death in melanoma cell lines through targeting of Mcl-1 and A1. PLoS One 7: e30821. 22292048 10.1371/journal.pone.0030821 PMC3265511

[34] Al Zaid Siddiquee K, Turkson J (2008) STAT3 as a target for inducing apoptosis in solid and hematological tumors. Cell Res 18: 254–267. 18227858 10.1038/cr.2008.18 PMC2610254

[35] Yang F, Van Meter TE, Buettner R, Hedvat M, Liang W, et al (2008) Sorafenib inhibits signal transducer and activator of transcription 3 signaling associated with growth arrest and apoptosis of medulloblastomas. Mol Cancer Ther 7: 3519–3526. 19001435 10.1158/1535-7163.MCT-08-0138 PMC2592687

[36] Kim BH, Jee JG, Yin CH, Sandoval C, Jayabose S, et al (2010) NSC114792, a novel small molecule identified through structure-based computational database screening, selectively inhibits JAK3. Mol Cancer 11: 36–48. 10.1186/1476-4598-9-36 PMC2830973 20149240

[37] Liu X, Guo W, Wu S, Wang L, Dai B, et al (2012) Antitumor activity of a novel STAT3 inhibitor and redox modulator in non-small cell lung cancer cells. Biochem Pharmacol 10: 1456–64. 10.1016/j.bcp.2012.02.010 PMC3391570 22387047

